# Particle-associated bacteria in seawater dominate the colony-forming microbiome on ZoBell marine agar

**DOI:** 10.1093/femsec/fiac151

**Published:** 2022-12-13

**Authors:** Anneke Heins, Jens Harder

**Affiliations:** Department of Molecular Ecology, Max Planck Institute for Marine Microbiology, Celsiusstr.1, D-28359 Bremen, Germany; Department of Molecular Ecology, Max Planck Institute for Marine Microbiology, Celsiusstr.1, D-28359 Bremen, Germany

**Keywords:** bacterioplankton, chemotaxis, cultivability, North Sea, phycosphere, sedimentation cone

## Abstract

Planktonic particle-associated bacteria comprise particle-attached and motile free-living cells. These groups were obtained by settlement in Imhoff cones. Dilution plating on marine agar 2216 (ZoBell marine agar) and microscopic counts indicated a cultivability of 0.7% (0.4%–1.2%) of bacteria in coastal seawater collected at Helgoland Roads, North Sea. Particle-associated bacteria presented a minority population in seawater, but had a larger cultivability of 25% (0.9%–100%) for populations collected by settlement of particles and 5.7% (0.9%–24%) for populations collected by filtration. Partial 16S rRNA gene sequences indicated that 84% of the cultured taxa were either enriched in particle-associated microbiomes or only found in these microbiomes, including *Sulfitobacter* and other *Rhodobacteraceae, Pseudoalteromonas, Psychromonas, Arcobacter* and many *Flavobacteriaceae*. Illumina-based 16S rRNA V3V4 amplicon sequences of plate communities revealed that nearly all operational taxonomic units had a cultivated and described strain in close phylogenetic proximity. This suggested that decades of strain isolation from seawater on ZoBell marine agar had achieved a very good coverage of cultivable genera abundant in nature. The majority belonged to particle-associated bacteria, complementing observations that abundant free-living seawater bacteria often require cultivation conditions closer to their natural habitat like liquid cultivation in oligotrophic medium.

## Teaser

Marine bacteria cultivable on plates are dominated by a minor population of planktonic bacteria that lives preferentially close to or on phytoplankton and particles.

## Introduction

The great plate count anomaly depicts the discrepancy between the direct microscopic count of all cells in aquatic habitats and the capacity to grow as colonies on solid agar media (Staley and Konopka 1985). The viable cells represented 0.1% to 1% of all cells and the authors hypothesized that the non-viable cells were different, probably being oligotrophic bacteria. Today, most cells of the planktonic bacterial community still remain uncultivated on plates (Joint et al. 2010, Steen et al. 2019), despite high-throughput approaches using several media and dilution series at environmental temperatures (Alejandre-Colomo et al. 2020). With liquid media, isolation experiments had observed cultivabilities of 35% (Hahnke et al. 2015) and 50% (Button et al. 1993). ZoBell´s marine agar has been the standard plate medium for the cultivation of marine bacteria for more than 60 years (Oppenheimer and ZoBell 1952). It is still among the media enabling the highest numbers of colony-forming units (CFUs) (Eilers et al. 2000, Hahnke and Harder 2013, Alejandre-Colomo et al. 2020).

Aquatic habitats are not homogeneous, they offer a range of niches in time and space, that is, close to particles (Caron et al. 1982) and phytoplankton (Stocker 2012). These niches support high diversity in aquatic microbiomes. Small, non-motile free-living bacteria constitute the large majority of the communities (Koch 2001, Morris et al. 2002, Lauro et al. 2009). They often have small genomes and a small substrate range (i.e. of polysaccharides) (Caron et al. 1982, Alldredge et al. 1986, Rappé et al. 2002, Simon et al. 2002, Lauro et al. 2009, Smith et al. 2013b, Rieck et al. 2015, Kappelmann et al. 2019). These traits likely result from genome streamlining, an adaptation to the oligotrophic marine environment with its micromolar substrate concentrations (Giovannoni et al. 2014). This physiology is in contrast to the more copiotrophic physiology of smaller populations of motile free-living and of particle-attached bacteria. Motile free-living bacteria have a chemotactic sensing and use one or more polar or lateral flagella to swim to phytoplankton and other particles (Lauro et al. 2009, Stocker and Seymour 2012). Particle-attached bacteria possess attachment capability and often gliding motility (Burchard 1980, Liao et al. 2015). Their close proximity to nutrient hot spots—phytoplankton and particles in the sea—enables high metabolic activity, making them significant microbial contributors to biochemical cycles (Smith et al. 1992, DeLong et al. 1993, Grossart et al. 2007, Ziervogel and Arnosti 2008, Lauro et al. 2009, Ziervogel et al. 2010, Pedler et al. 2014). These particle-associated bacteria are a minority of the bacterioplankton in marine and freshwater habitats. In previous publications, particle-attached cell populations ranged from low percentages such as 0.1%–4% (Alldredge et al. 1986) to up to 32% (Ghiglione et al. 2007). Motile populations ranged from 10% to 80% (Mitchell et al. 1995; Grossart et al. 2001, Stocker and Seymour 2012). It was suggested that their adaptation to nutrient-rich particles implies a better cultivability, especially on solid media (Hahn et al. 2004).

In this contribution, we experimentally test the hypothesis that viable marine bacteria on plates are dominated by particle-associated bacteria. So far, sequential filtration has been applied to separate particles from free-living bacteria, mainly by 3.0-µm filters (Ayo et al. 2001, Crespo et al. 2013, Bižić-Ionescu et al. 2015). It does not separate non-motile from motile free-living bacteria. To obtain an enrichment of motile free-living bacteria, we have recently introduced Imhoff´s sedimentation cones that allow motile, chemotactic bacteria to follow the settling phytoplankton and other particles by swimming to the bottom of the cone (Heins et al. 2021b). This separation resulted in a top water fraction enriched in non-motile free-living bacteria and a bottom fraction highly enriched in particles, particle-attached bacteria and chemotactic free-living bacteria. In combination with sequential filtration and centrifugation, we used enrichment factors (EFs) derived from relative read abundances of 16S rRNA gene amplicons in subpopulations to classify aquatic microorganisms into five groups: (a) fragile (filtration-sensitive) or (b) rigid free-living bacteria smaller than 3 µm, (c) non-sinking large bacteria and bacterial aggregates, (d) swimming chemotactic particle-associated bacteria and (e) particle-attached bacteria (Heins et al. 2021b). The enrichment factors reflect the distribution of taxa over the niches in aquatic habitats, as each microorganism spends a part of its life free-living in the sea, as offspring or in search of new food sources (Iversen and Ploug 2010). The separation of populations in sediment cones enabled the separation of free-living bacteria into non-motile and motile bacteria, a prerequisite to study oligotrophic and copiotrophic subpopulations and to verify experimentally the hypothesized prevalence of particle-associated bacteria among the cells growing on ZoBell marine agar.

## Material and Methods

### Sampling

Seawater was sampled 1 m below the surface at the long-term ecological research site Helgoland Roads (54°11’03’N, 7°54’00’E) during a diatom-dominated phytoplankton spring bloom in April and May 2018 (Heins et al. 2021b). Five sampling time groups were chosen covering pre-bloom condition (groups 1 and 2) and bloom conditions with the first and second chlorophyll *a* maxima and an intermediate period (groups 3, 5 and 4, respectively, Table S4). The seawater was stored at 4°C in 10-L containers and was processed within 2 h after sampling, starting by gently inverting the resuspended particles. Additionally, plankton net catches were obtained with a pore size of either 20 or 80 µm.

### Seawater fractionation and sample plating

Unprocessed seawater samples were diluted in a 10-fold series with artificial sterile seawater (Winkelmann and Harder 2009) prior to inoculation. A plate inoculum of 200 µl was distributed with a sterile, one-way inoculation loop using a plate rotator. The medium was ZoBell marine agar (marine agar 2216, Difco Laboratories, Detroit, MI, USA).

For the separation by sequential filtration, 500 ml of seawater was filtered through 10-µm (filter code: TCTP) and 3-µm (filter code: TSTP) pore-sized filters, resulting in two particle-attached and one free-living fraction from the two retentates "F_10 µm" and "F_3 µm", as well as the 3-µm filtrate "F_0.2 µm", respectively. "F_0.2 µm" comprised all cells with a size of 0.0 to 3 µm, but only the cells of the retentate on a 0.2-µm filter (filter code: GTTP) were counted, resulting potentially in an overestimation of the cultivability of the free-living cells. Also, seawater bacteria were counted after direct filtration onto a 0.2-µm filter. All filters were composed of a hydrophilic polycarbonate membrane (Millipore, Darmstadt, Germany) and had a diameter of 47 mm, with an effective filter diameter of 39 mm and an effective filter area of 1195 mm^2^. Cells of the particle fractions were recovered by rinsing the filters repeatedly with 3 ml of artificial seawater and collecting the particles in a sterile Petri dish. One ml of the resuspension was homogenized gently by shear force using sterile pestles and tubes (Bel-Art ProCulture micro-tube homogenizers, Thermo Fisher Scientific Inc., Schwerte, Germany). All fractions were diluted and plated as aforementioned.

A Rotina 35R centrifuge (Hettich, Tuttlingen, Germany) was used to obtain a particle fraction (pellet) and a free-living fraction (supernatant) from 50 ml of seawater by forced gravitation, applying 4890 x *g* for 10 min. The pellet was removed with a pipette. Homogenization and plating were performed as aforementioned. This method had a limited separation capacity (Heins et al. 2021b).

Imhoff sedimentation cones (Brand Scientific, Wertheim, Germany) were used to separate 1 L of seawater by natural gravitation. After 3 h of settlement, particles at the bottom of the cone were removed through a stopcock and dispersed with a micro homogenizer. Both the top fraction (upper water layer, free-living fraction) and the bottom fraction (particle-associated bacteria) were diluted and plated as aforementioned.

Some harvested bottom fractions were further separated. After resuspension in 50 ml of artificial seawater (ASW) they were centrifuged with 4890x *g* for 10 min, resulting in a free-living motile (supernatant) and a particle-attached bottom fraction.

The particle-rich samples retrieved from the 20- and 80-µm pore-sized plankton net catches were homogenized, diluted and plated as described above.

### DADA2 analyses of the microbial communities in inocula

Bacterial communities in the inocula have previously been characterized (Heins et al. 2021b). The reads (accessible in the European Nucleotide Archive project PRJEB41742) were reanalyzed using the DADA2 pipeline (version 1.16.0) (Callahan et al. 2016) with the standard filtering parameters: maxN = 0, truncQ = 2, rm.phix = TRUE and maxEE = 2. With the "learnErrors" method 103 431 192 total bases in 454 453 reads from six samples were used for learning the error rates of R1 (forward reads), and 104 301 605 total bases in 454 453 reads from six samples were used for learning the error rates of R2 (reverse reads). A core sample inference algorithm with the parameters OMEGA_A = 1e-40, OMEGA_C = 1e-40 and BAND_SIZE = 16 was applied to the filtered and trimmed sequences. Denoised forward and reverse reads were merged only if they had an overlap of at least 12 bases. The "removeBimeraDenovo()" function was used to remove chimeras. Less than 1% of the merged sequence variants were determined to be chimeric. Taxonomy was assigned to the amplified sequence variant (ASV) table without chimeras based on the Silva database (v. 138.1).

### Partial 16S rRNA gene sequencing of individual colonies

Colonies were imaged with a Nikon Coolpix AW110 camera (Nikon, Chiyoda, Japan) and then a small amount of each colony was transferred into 20 µl of PCR-grade water using sterile 1-µl pipette tips. The cell material was dispensed by twisting the pipette tip between the fingertips for 30 s. Cells were broken up in five freeze and thaw cycles iterating between –20 and 4°C. One µl of the broken cell suspension contained approximately 50 ng of DNA and served as a template for partial 16S rRNA gene amplification, together with 15 µl of 2x High-Performance GoTaq® G2 DNA Polymerase Master Mix (Promega, Madison, WI, USA), 0.3 µl of a 100 pmol µl^−1^ working solution of the reverse primer 907R (5′-CCGTCAATTCCTTTRAGTTT-3′), 0.05 µl of a 100 pmol µl^−1^ working solution of the forward primer 27f-YM (5′-AGAGTTTGATYMTGGCTCAG-3′) and 13.65 µl of PCR-grade water (Muyzer et al. 1995, Frank et al. 2008). The cycler program began with a 4-min denaturation at 94°C, followed by 35 cycles of a 1-min denaturation at 94°C, 1 min of annealing at 55°C and a 3-min elongation at 72°C, with a final elongation for 10 min at 72°C. After purification with Sephadex G-50 superfine columns (Sigma-Aldrich, Taufkirchen, Germany), the amplicon was sequenced with the reverse primer 907R using a 3130xl Genetic Analyzer (Applied Biosystems, Foster City, CA, USA). Sequences were trimmed with Sequencher 4.6 (Gene Codes, Ann Arbor, MI, USA) and compared against the databases NCBI, the living tree project (LTP, Ludwig et al. (2021)) and SILVA 138.1 (Pruesse et al. 2012) for strain affiliation. The sequences were deposited in the NCBI database under the accession numbers OM316820-OM317558.

### 16S rRNA gene amplicon sequencing of plate microbiomes

For Illumina amplicon sequencing, biomass of 407 dilution plates of individual samples was harvested from four seawater samples, 11 samples enriched in free-living bacteria and 31 samples enriched in particle-associated bacteria (Table S4). For each sample, only the plates with inocula of the three highest dilutions that showed growth were harvested, containing 1–15, 10–100 and 100–300 CFUs or a lawn of coalesced colonies, respectively. Plates were wetted with artificial seawater and sterile one-way inoculation loops were used to collect the suspensions of all dilution plates in a centrifugation tube. After centrifugation, the biomass was weighted and resuspended in phosphate buffer to 150 mg of biomass ml^−1^. DNA was extracted from 15 mg of biomass with a MP Biomedicals™ FastDNA™ SPIN Kit for soil samples according to the manufacturer´s protocol (Thermo Fisher Scientific). DNA concentration, fragment length analysis and amplification of the hypervariable V3V4 region of the bacterial 16S rRNA gene were performed as described in Heins et al. (2021b). Equimolar amplicon pools were sequenced on an Illumina HiSeq2500 in rapid mode with a 2 × 250 bp paired-end run performed by the Max Planck Genome Centre, Cologne, Germany (https://mpgc.mpipz.mpg.de/home/).

Next, 5.2 million 16S rRNA reads were paired with BBmerge v. 37.82 applying default settings without mismatches in the overlapping region (Bushnell et al. 2017). A total of 4.3 million merged reads were de-multiplexed and quality trimmed using MOTHUR (Schloss et al. 2009). The command "trim.seqs" was used with the setting of a minlength = 300, maxambig = 0, maxhomop = 8, allfiles = T and checkorient = T. The resulting high-quality merged sequences (3.9 million) were classified with the SILVAngs pipeline (Quast et al. 2013) using SILVA release version 138.1, SINA version 1.2.10 and VSEARCH version 2.15.1. We used a minimum alignment length of 150, a minimum quality score of 30 (minimum length of a sequence/reads) and a similarity threshold of 0.99 for the creation of operational taxonomic units (OTUs). Reads that did not produce a BLAST (v. 2.2.30+) hit, for which the sum of the sequence identity and the alignment coverage divided by 2 was higher or equal to 93%, were assigned and combined as reads with "no relative" (SILVAngs 2015). In total, 3 876 908 sequences were classified and analyzed after processing, with a minimum of 35 921 and maximum of 90 908 per sample. Read abundance was normalized for each sample using R and the "decostand()" function (method = total) of the vegan package (Oksanen et al. 2020). The large number of Illumina reads likely supported the detection of taxa that underwent substrate-induced cell death or only formed microcolonies. We defined a growth threshold, based on the maximum relative read abundance of the most abundant SAR11 clade. Members of the SAR11 clade constitute the majority of the bacterioplankton in coastal areas (more than 30%) (Morris et al. 2002, Eiler et al. 2009, Teeling et al. 2012, Teeling et al. 2016), but do not grow to visible colonies on plates (Rappé et al. 2002). Raw reads of all 46 samples were deposited at the European Nucleotide Archive and are accessible under the number PRJEB50169.

### Statistical evaluation and visualization

Statistical analyses, data transformations and graphing were performed in R studio (R Core Team 2014). Statistical evaluation of the CFU communities between the methods was performed with a Permutational Multivariate Analysis of Variance (PERMANOVA) using distance matrices from the "vegan" package, with the "vegdist()" function and the "method = bray" setting (Oksanen et al. 2020). This analysis was followed by a pairwise comparison using the "pairwise.perm.manova()" function and the "method = Euclidian" setting. The packages ComplexHeatmap (Gu et al. 2016), circlize (Gu et al. 2014), picante (Kembel et al. 2010), rioja (Juggins 2020), colorspace (Zeileis et al. 2009) and dplyr (Wickham et al. 2018) were used for further data visualization and transformation.

## Results and Discussion

### Cultivability of microbial populations

Seawater was collected during a diatom-dominated phytoplankton spring bloom at Helgoland Roads off Helgoland, an offshore island in the German Bight of the southern North Sea. Filtration, centrifugation and gravitation in Imhoff cones were applied to obtain seawater fractions enriched in subpopulations of free-living and of particle-associated bacteria. Particle-containing bottom fractions of sedimentation cones were sometimes centrifuged to separate the motile free-living bacteria from particle-attached cells. Total cell counts (Table 1) showed over the duration of the 2018 North Sea spring season a bacterioplankton bloom (Heins et al. 2021b). In the microscopic view, less than 1% of all bacteria were attached to particles (Heins et al. 2021b). Seawater and fractions thereof were serial diluted with artificial seawater and then inoculated on ZoBell marine agar. The concentration of CFUs (always referring to seawater) increased with rising total cell counts per ml seawater (Table 1). We defined cultivability as colonies larger than 1 mm in diameter (ml sample)^−1^ divided by the DAPI-stained (DNA-containing) cells (ml sample)^−1^. Seawater-inoculated plates showed, with 0.5% to 0.7%, a cell cultivability in the range of previous observations for unprocessed seawater samples from the same sampling site (Helgoland Roads) (Gerdts et al. 2004, Alejandre-Colomo et al. 2020, Heins et al. 2021a). Although free-living fractions contained less particles than unfractionated seawater, a similar cultivability was observed, ranging from 0.4% to 1.2%. Overall, the free-living populations contributed most CFUs; only a small portion was obtained from the particle-associated fractions. These observations are of limited value with respect to the habitat of the cells, because particle-attached cells spend a certain time of their lives as free-living cells in search of new particles.

**Table 1.** Total cell counts, colony-forming units (CFUs) and cultivability of microbiomes in seawater and fractions thereof.

	Sample	12.4.2018	18.4.2018	25.4.2018	08.5.2018	22.5.2018
Total cell counts (x10^3^ cells ml^−1^)	SW	605 ± 75	847 ± 94	1100 ± 90	1441 ± 205	2472 ± 250
	F_0.2 µm	438 ± 22	469 ± 52.4	738 ± 62	922 ± 94	1678 ± 192
	F_3 µm	1.6 ± 0.7	1.5 ± 1.1	1.4 ± 0.9	1.4 ± 0.3	4.5 ± 1.2
	F_10 µm	1.1 ± 0.6	1.4 ± 2	1.6 ± 2.2	1.6 ± 2	5.8 ± 6.1
	C_FL	383 ± 58	598 ± 102	808 ± 113	nd	nd
	C_PA	13.1 ± 2.3	11.1 ± 6.2	20.9 ± 5.4	nd	nd
	SC_TF	562 ± 79	661 ± 121	777 ± 94	1011 ± 153	1767 ± 203
	SC_BF	1.5 ± 0.2	1.1 ± 0.8	0.4 ± 0.2	1.3 ± 0.4	3.5 ± 0.7
	SC_BF_FL	1.5 ± 0.5	nd	0.3 ± 0.2	nd	nd
	SC_BF_PA	0.3 ± 0.0	nd	0.1 ± 0.1	nd	nd
CFUs ml^−1^	SW	nd	4353 ± 1919	5433 ± 2340	10 533 ± 1841	12 210 ± 4575
	F_0.2 µm	2343 ± 1091	1963 ± 809	5607 ± 1996	8693 ± 2728	15 740 ± 5371
	F_3 µm	24 ± 2	13 ± 7	50 ± 13	46 ± 10	229 ± 108
	F_10 µm	264 ± 199	41 ± 22	50 ± 10	99 ± 58	355 ± 142
	C_FL	nd	3040 ± 1358	3040 ± 1358	nd	nd
	C_PA	nd	161 ± 125	141 ± 40	nd	nd
	SC_TF	nd	3440 ± 427	5933 ± 2541	8827 ± 3153	20 340 ± 6646
	SC_BF	13 ± 1	263 ± 166	50 ± 37	47 ± 13	222 ± 9
	SC_BF_FL	nd	54 ± 15	243 ± 75	nd	nd
	SC_BF_PA	nd	10 ± 4	117 ± 85	nd	nd
Culturability (%)	SW	nd	0.5	0.5	0.7	0.5
	F_0.2 µm	0.5	0.4	0.8	0.9	0.9
	F_3 µm	1.5	0.9	3.6	3.3	5.1
	F_10 µm	24.0	2.9	3.1	6.2	6.1
	C_FL	nd	0.5	0.4	nd	nd
	C_PA	nd	1.5	0.7	nd	nd
	SC_TF	nd	0.5	0.8	0.9	1.2
	SC_BF	0.9	23.9	12.5	3.6	6.4
	SC_BF_FL	nd	nd	81.1	nd	nd
	SC_BF_PA	nd	nd	117.2	nd	nd

C_FL/PA: centrifugation-derived free-living ( = supernatant) and particle-attached ( = pellet) fraction; nd: not determined; F_10 µm, F_3 µm, F_0.2 µm: sequentially filtered fractions of the respective size range >10 µm, 10–3 µm and <3 µm; SC_BF/TF: sedimentation cone bottom and top fraction after 3 h of sedimentation; SC_BF_FL/PA: sedimentation cone bottom fraction after 3 h of sedimentation, resuspension and separation by centrifugation; SW: seawater.

The particle-enriched fractions had a higher cultivability than fractions enriched in free-living bacteria (Table 1). Microbiomes associated with particles of >10 µm had, with 24%, the highest cultivability. Also, the particle-containing bottom fraction of sedimentation cones showed a high cultivability. This not only included the particle-attached bacteria, but also the free-living motile bacteria collected in the supernatant after centrifugation of the bottom fraction (Table 1), indicating that many motile particle-associated and particle-attached bacteria grew readily on plates.

**Figure 1.**
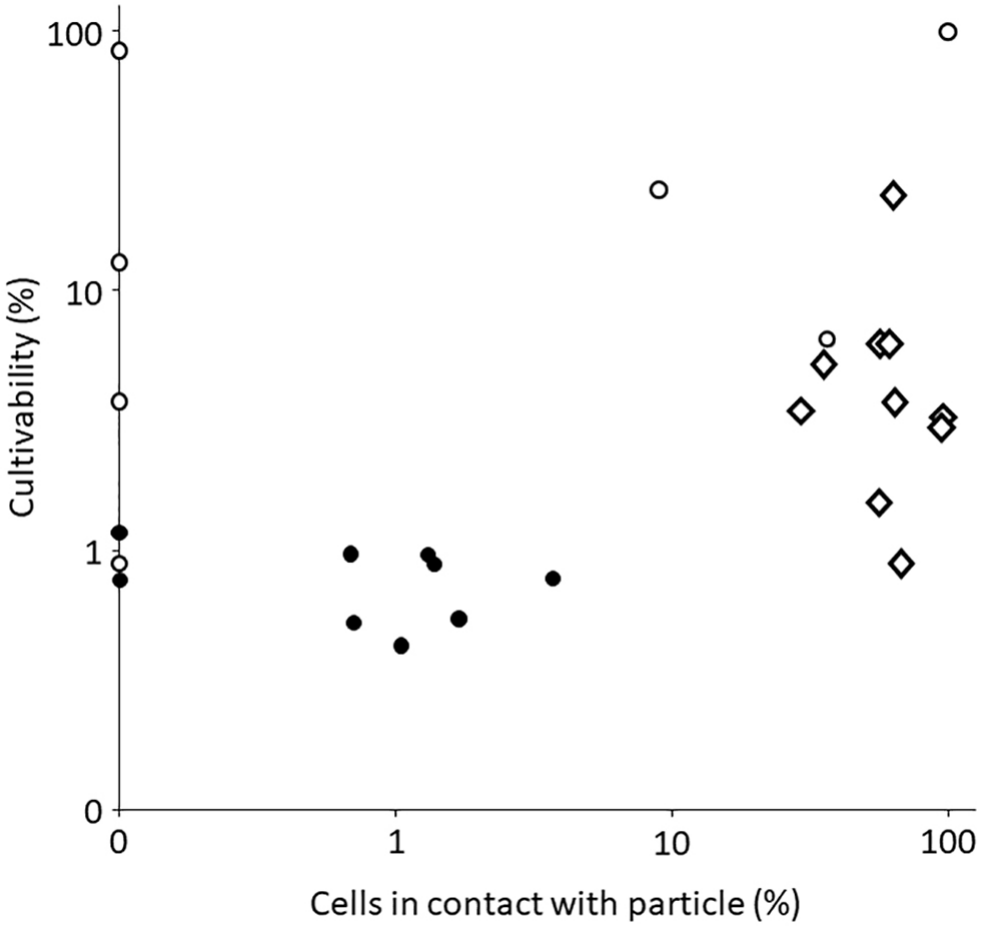
Relative population of particle-attached bacteria observed by microscopy in sample microbiomes and cultivability of these microbiomes on 2216 agar plates. Samples were taken during a phytoplankton spring bloom off Helgoland in 2018. Cell counts of particle-associated cells were from Heins et al. (2021b). Microbiomes were enriched in free-living bacteria (full circle) or particle-attached bacteria (by filtration, open diamond) or particle-associated bacteria (by settlement in cones, open circle).

Microscopy provided not only the means to count cells on filters, but also to count the portion of cells in direct contact with algae or transparent exopolymeric particles (Heins et al. 2021b). The cultivability increased with a higher percentage of cells in direct contact with particles, from 0.67% ± 0.23% (n = 15) for seawater and free-living fractions to 5.7% ± 6.7% (n = 10) for cells washed off 3- and 10-µm filters and 25% ± 38% (n = 6) for cells that had settled together with particles. In a single experiment, particle-associated cells were separated from particles by centrifugation and had a cultivability of 81% (Table 1 and Fig. 1). Overall, the concentration of particle-attached cells in seawater was larger than that of CFUs, sufficient to explain all the CFUs in seawater samples. The observations also indicated that not all particle-attached cells form colonies on ZoBell marine agar (Table S1).

### Diversity of individual colonies

To further support a prevalence of particle-associated bacteria among the colonies grown on ZoBell marine agar, we characterized the diversity of cells growing on plates, performing partial 16S rRNA gene sequencing of individual colonies and of whole plate communities using Sanger sequencing and amplicon library sequencing on the Illumina platform, respectively.

**Figure 2.**
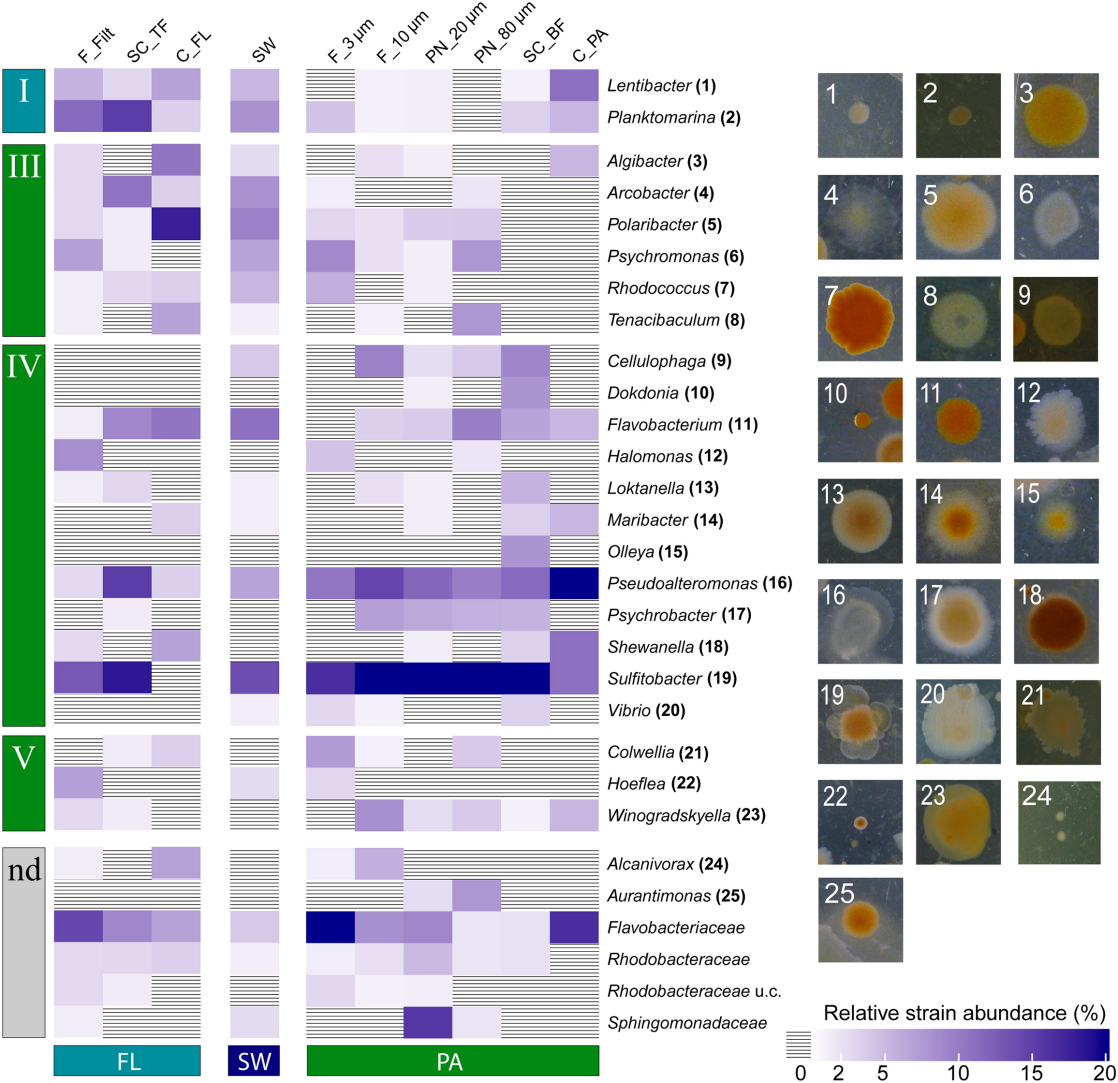
Relative abundance within CFU microbiomes and colony phylogeny of the 29 most abundant genera (≥7 CFUs genus^−1^). CFUs grown on 2216 plates were individually sampled and identified using Sanger sequencing. Roman numerals indicate culture-independent free-living (I) and particle-associated (III-V) bacterial groups, as described in Heins et al. (2021b). I: free-living bacteria smaller than 3 µm; III: large, filamentous or aggregate (rosette)-forming bacteria; IV: particle-associated bacteria living in both modes (motile free-living and particle-attached); V: particle-attached bacteria; nd: not defined (not classified in one of the groups). C_FL/PA: centrifugation-derived free-living (= supernatant) and particle-attached (= pellet) fraction; F_10 µm, F_3 µm, F_0.2 µm: sequentially filtered fractions of the respective size range >10, 10–3 and <3 µm, respectively; PN_20 µm/ 80 µm: plankton net fractions caught with a net size of 20 or 80 µm; SC_BF/TF: sedimentation cone bottom and top fraction after 3 h of gravitational settlement; SW: seawater.

Individual, morphologically often diverse colonies from plates with the smallest inoculum yielded 739 sequences affiliated to 106 genera or families. The most abundantly sequenced genera were isolated from all fractions, an indication that each bacterium, even particle-attached bacteria, spends a certain time free-living in the sea (Iversen and Ploug 2010) (Fig. 2, Table S2). Following the lifestyle classification based on seawater fractionation and enrichment factors (Heins et al. 2021b), within the abundantly present genera (≥7 strains), only *Planktomarina* and *Lentibacter* were classified as members of the free-living bacteria (group I). Both bacteria formed small colonies (Fig. 2). The majority of colonies was affiliated with 21 genera identified as particle-attached or particle-associated bacteria: *Algibacter, Tenacibaculum* and *Psychromonas* were members of a group that can be enriched by particle filtration (group III); group IV comprised particle-associated bacteria that were either particle-attached or free-swimming, like *Maribacter, Cellulophaga, Pseudoalteromonas, Shewanella, Sulfitobacter* and *Vibrio. Colwellia, Hoeflea* and *Winogradskyella* represented the strongly particle-attached group (group V) (Fig. 2). These particle-associated bacteria formed large colonies on plates, with the exception of *Hoeflea* and *Dokdonia*. Many are known to attach to phyto- and zooplankton or are part of the phycosphere (Mestre et al. 2017, Seymour et al. 2017, Heins et al. 2021b). In addition, cells of these genera pass through a 3-µm pore-sized filter in their free-living stage, which explains the occurrence in the free-living, particle-free fraction. These results support the hypothesis that particle-associated bacteria dominate the CFU microbiome on ZoBell marine agar.

Seawater and subfraction microbiomes used in this study were previously described in a cultivation-independent study (Heins et al. 2021b). We used the reads to perform a DADA2 analysis to enhance the taxonomic resolution of the affiliation from genus to ASVs of the highly variable V3V4 region of the 16S rRNA gene (Table S3). These ASVs present amplicons of 99% sequence identity, whereas in the Silva classification the affiliation of a sequence to a genus can be based on an identity threshold as low as 93%. Overall, the DADA2 analysis of 40 inocula communities with 3 236 537 reads identified 22 603 ASVs in total, of which we considered 10 461 partial 16S rRNA gene variants with at least two reads and at least a relative read abundance of 0.01% in one sample in the following analysis. The enrichment factor analysis of the ASVs confirmed the free-living lifestyle of well-known planktonic bacteria including *Ca*. Actinomarina, *Formosa*, the flavobacterial North Sea groups NS2, NS3a, NS4 and NS5, SAR406, *Planktomarina*, AEGEAN-169, SAR11 clade I, OM43 and SAR86 and SAR92.

Of 739 CFU sequences, 95% affiliated with at least 98.7% identity to one of 249 ASVs observed in the inocula communities from Helgoland. Using enrichment factors of the ASVs (Heins et al. 2021b), we determined the preferred habitat of the 249 ASVs that represented the colony-forming cells. Only 21 ASVs were assigned as free-living bacteria, whereas 124 ASVs were more enriched in particle-associated fractions. Nineteen ASVs were nearly equally distributed (EF values between 0.8 and 2.0). According to this analysis, *Leeuwenhoekiella, Subsaxibacter, Brevundimonas, Planktomarina, Planktotalea, Limnobacter* and *Acidovorax* were free-living bacteria. Free-living as well as particle-associated ASVs were present in *Flavobacterium, Polaribacter, Sulfitobacter, Loktanella, Pseudoalteromonas, Shewanella, Alcanivorax* and *Psychrobacter*. Although some of these genera have a rather broad taxonomic breadth, it suggests that a genus can include free-living and particle-associated species. Eighty-five ASVs were rare ASVs not detected in seawater, which is a prerequisite for the calculation of an enrichment factor. This finding is also evidence of an access to rare species by fractionation of seawater.

### Diversity of plate microbiomes

The characterization of CFU microbiomes by Illumina-based partial 16S rRNA gene amplicon sequencing has recently more often been applied, that is, with CFUs from a raccoon (Junkins and Stevenson 2021) and from tropical sediment (Demko et al. 2021). We used the technology to cover the (whole) diversity on plates and to use the method´s sensitivity to identify bacteria of small colonies that were not visible to the naked eye. A dilution series yielded the biomass of three plates with different inocula population sizes, containing statistically 1–15, 10–100 and 100–1000 CFUs. Series of consecutive days were combined to obtain finally, for five time points during the spring bloom, 46 samples with the biomass of 407 plates (Table S4). We observed plates—90 mm in diameter—with up to 300 visible colonies, but sometimes a lawn of bacteria. In this lawn, bacterial interactions may have modified the growth through benefiting or inhibiting factors. To exclude these biological influences, the next generation of experiments linking CFU microbiomes to 16S rRNA amplicon sequencing has to aim at thousands of individual colonies per sample grown within distance of each other.

The definition of a growth threshold was guided by the abundance of a genus in seawater and knowledge of its cultivability on plate media. We selected the most abundant SAR11 OTU as the threshold to delimit successful growth. The SAR11 clade is the most abundant taxon in the photic zone of the ocean and its members do not grow on solid media (Morris et al. 2002, Rappé et al. 2002, Eiler et al. 2009, Teeling et al. 2012, 2016). In the Illumina amplicon dataset, the most abundant SAR11 OTU had relative read abundances of 5 × 10^−5^ in a sample. Hence, an OTU relative read abundance of >1×10^−4^ was evaluated as successful growth.

The dataset of 3.9 million reads included, in total, 65 294 OTUs with a sequence identity of over 99%. These were classified into 592 taxa based on a threshold of >93% identity to a reference sequence in the Silva 138.1 dataset. Only 512 reads (0.01%) had no reference sequence and, therefore, were listed in a group of "no relatives". This number is very low in comparison with the cultivation-independent direct analyses of Helgoland samples that resulted in 0.5% to 2.6% of "no relatives" for seawater and up to 19% in particle fractions (Heins et al. 2021b).

**Figure 3.**
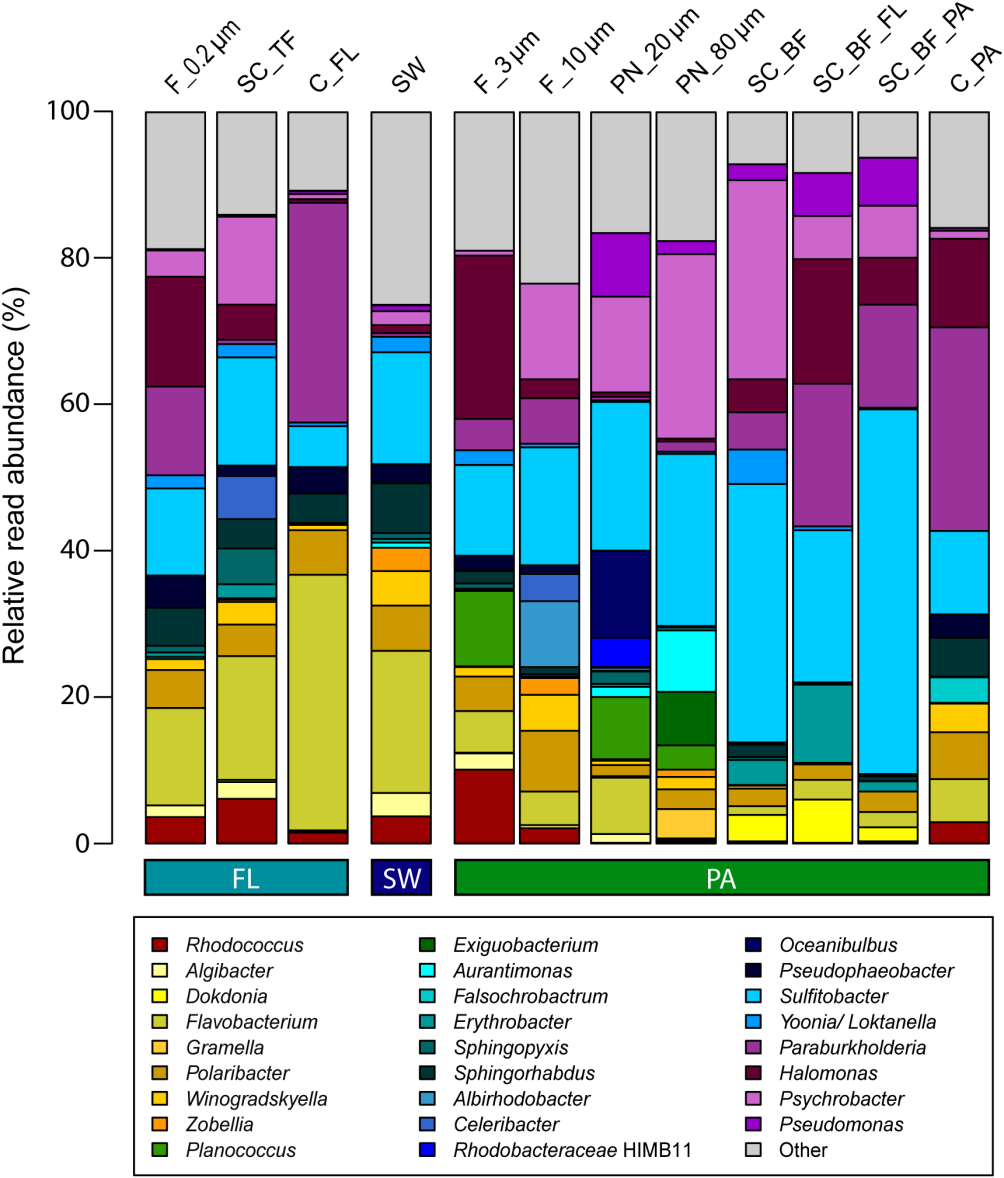
Microbial communities on plates investigated by partial 16S rRNA amplicon sequencing: relative read abundance of genera that had in the Illumina reads, in at least one sample, a relative abundance of ≥3%. Samples were taken during a phytoplankton spring bloom off Helgoland in 2018. Color scales indicate higher order affiliation: red: *Actinobacteria*, yellow: *Flavobacteriia*, green: *Firmicutes*, blue: *Alphaproteobacteria*, purple: *Gammaproteobacteria*. C_FL/PA: centrifugation-derived free-living (= supernatant) and particle-attached (= pellet) fraction; F_10 µm, F_3 µm, F_0.2 µm: sequentially filtered fractions of the respective size range >10, 10–3 and <3 µm; PN_20 µm/80 µm: plankton net fractions caught with a pore size of 20 or 80 µm; SC_BF/TF: sedimentation cone bottom and top fraction after 3 h of sedimentation; SC_BF_PA/FL: sedimentation cone bottom fraction after 3 h of sedimentation, resuspension and separation by centrifugation into a free-living (FL) and particle-attached (PA) fraction; SW: seawater.

On the genus level, most reads affiliated with *Sulfitobacter*, an alphaproteobacterial genus of the *Rhodobacteraceae* and a motile member of the phycosphere and particle microbiomes (Seymour et al. 2017) (Fig. 3). Other abundant alphaproteobacterial OTUs were affiliated (with decreasing read numbers) to *Sphingorhabdus, Pseudophaeobacter*, members of the *Yoonia/Loktanella* twin genera clade (Wirth and Whitman 2018), *Erythrobacter*, "Oceanibulbus" (now *Sulfitobacter* (Liu et al. 2017)),*Albirhodobacter, Celeribacter* and *Hoeflea* (Fig. 3). The *Gammaproteobacteria* were less diverse and only four genera were frequently present across samples. These were, in decreasing order of their relative read abundance, *Psychrobacter*, members of the *Burkholderia-Caballeronia-Paraburkholderia* clade,*Halomonas* and *Pseudomonas. Psychrobacter* was highly abundant in the filtered and plankton net fractions >10 µm and in the sedimentation cone fractions (Fig. 3).

Within the phylum *Bacteroidetes*, the genetically broad genus *Flavobacterium* was most abundant and selectively enriched from seawater and free-living fractions. All other abundant flavobacterial genera, that is, *Polaribacter, Winogradskyella, Algibacter, Cellulophaga, Dokdonia* and *Zobellia*, are known as particle-associated or particle-attached bacteria. *Gramella* was found in high abundance (>3%) on plates inoculated from the >80 µm plankton net fraction (Fig. 3), as expected from the cultivation-independent characterization of the samples (Heins et al. 2021b).

Overall, the CFU community compositions of fractions were significantly different from each other (*P* < 0.001, Permanova, Table S5), especially between the plankton net and sedimentation cone bottom fractions—both contain highly enriched particle-associated communities—in comparison with the filtered free-living and small (3–10 µm) particle fractions (*P* < 0.05, pairwise Manova, Table S6). CFU seawater communities were similar to free-living community fractions (both the 0.2 to 3 µm fraction of sequential filtration and the top fraction of the sedimentation cones) (Table S6). These differences coincide with the variations observed directly on the fractions in a cultivation-independent community characterization (Heins et al. 2021b).

Quantification of such amplicon data has to be considered with some cautiousness. Besides the stochastic effect in the dilution series, rRNA operon copy numbers of strains and biomass variations per colony may have caused differences between both datasets (e.g. *Lentibacter* and *Planktomarina* formed only small colonies). Furthermore, taxon-specific biases of the sequencing technology further modulate the observed read abundance of individual taxa (Aird et al. 2011, Oyola et al. 2012).

### Comparison with the database of cultivated bacteria

**Figure 4.**
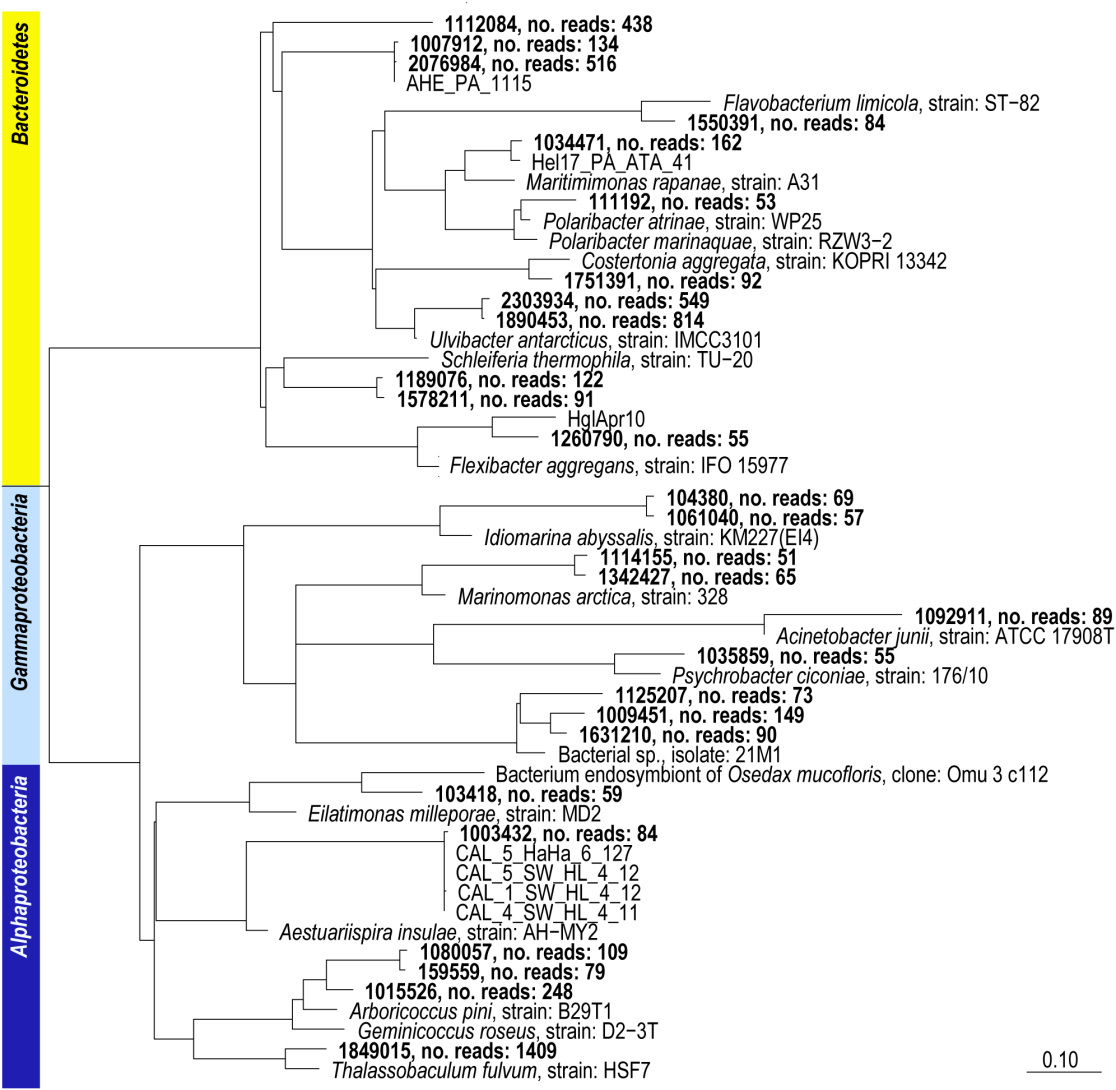
Phylogenetic position of OTUs that grew on plates and had an identity of <95% (bold letters) to the sequences of microorganisms present in the Bacterial and Archaeal 16S Ribosomal RNA RefSeq Targeted Loci Project (NCBI), based on maximum likelihood analyses. No. reads: total number of reads affiliating with an OTU in this study (out of 3.9 million reads in total). The scale bar indicates 10% sequence divergence.

The OTU classification used in this study referred to a database including sequences of as-yet uncultivated strains (Silva 138.1). In addition, we analyzed all ASVs of the whole dataset and identified 1336 ASVs (99% identical in V3V4 sequence) with read abundances at least twice of the one of the most abundant SAR11 OTU. These ASVs were compared with the Bacterial and Archaeal 16S Ribosomal RNA RefSeq Targeted Loci Project (PRJNA33175) of microorganisms in culture, available from NCBI. Most of the 1336 ASVs had an identity of 98% or more to a validly described species. Only 42 ASVs had, according to a BlastN analysis, an identity of below 95% (Fig. 4).

The fact that almost all ASVs had a related described species or strain is an indication that the isolation of planktonic marine bacteria on plates in the last few decades has covered nearly all cultivable genera abundant in nature, based on the resolution of partial 16S rRNA genes. This observation coincides with large-scale cultivation experiments. Using MALDI-ToF and full length 16S rRNA genes, Alejandre-Colomo et al. (2020) obtained 13 novel genera and 143 novel species within 3652 strains obtained from Helgoland seawater samples over a spring season. High diversity on the species level can be expected, but the diversity analyses of closely related species and of strains of a species are beyond this study and require marker genes with a higher taxonomic resolution or genome information (Žure et al. 2017, Delmont et al. 2019).

## Conclusion

Sedimentation cones (Heins et al. 2021b) were used to separate bacterioplankton into free-living non-motile bacteria and particle-associated bacteria comprising free-living motile and particle-attached bacteria. The particle-associated bacteria had a larger cultivability on ZoBell marine agar. In contrast to free-living planktonic bacteria, particle-associated bacteria encounter in nature more variable environmental conditions, that is, transiently high substrate concentrations, which are occurring locally for a short time when algae lyse (Seymour et al. 2017). These conditions coincide with the millimolar substrate concentrations in plate media. By contrast, genome-streamlined free-living bacteria lack defense systems (i.e. the PII system to inhibit the influx of ammonium) (Smith et al. 2013a, Alejandre-Colomo et al. 2021). Our diversity analyses suggest that cultivation on plates will still increase the number and diversity of species, however, most planktonic genera that are cultivable on ZoBell´s marine agar plates may already be represented by a strain in culture.

## Supplementary Material

fiac151_Supplemental_Files

## References

[bib65] Aird D , Ross MG, Chen W-S et al. Analyzing and minimizing PCR amplification bias in Illumina sequencing libraries. Genome Biol. 2011;12:1–14. 10.1186/gb-2011-12-2-r18 PMC3188800 21338519

[bib70] Alejandre-Colomo C , Francis B, Viver T et al. Cultivable *Winogradskyella* species are genomically distinct from the sympatric abundant candidate species. ISME Communications. 2021;1:51. 10.1038/s43705-021-00052-w PMC9723794 36747039

[bib4] Alejandre-Colomo C , Harder J, Fuchs BM et al. High-throughput cultivation of heterotrophic bacteria during a spring phytoplankton bloom in the North Sea. Syst Appl Microbiol. 2020;43:126066. 32019686 10.1016/j.syapm.2020.126066

[bib15] Alldredge AL , Cole JJ, Caron DA. Production of heterotrophic bacteria inhabiting macroscopic organic aggregates (marine snow) from surface waters. Limnol Oceanogr. 1986;31:68–78.

[bib32] Ayo B , Unanue M, Azua I et al. Kinetics of glucose and amino acid uptake by attached and free-living marine bacteria in oligotrophic waters. Mar Biol. 2001;138:1071–6.

[bib34] Bižić-Ionescu M , Zeder M, Ionescu D et al. Comparison of bacterial communities on limnic versus coastal marine particles reveals profound differences in colonization. Environ Microbiol. 2015;17:3500–14. 24674021 10.1111/1462-2920.12466

[bib23] Burchard RP . Gliding motility of bacteria. Bioscience. 1980;30:157–62.

[bib43] Bushnell B , Rood J, Singer E. BBMerge – Accurate paired shotgun read merging via overlap. PLoS One. 2017;12:e0185056. 29073143 10.1371/journal.pone.0185056 PMC5657622

[bib6] Button DK , Schut F, Quang P et al. Viability and isolation of marine bacteria by dilution culture: theory, procedures, and initial results. Appl Environ Microbiol. 1993;59:881–91. 16348896 10.1128/aem.59.3.881-891.1993 PMC202203

[bib38] Callahan BJ , McMurdie PJ, Rosen MJ et al. DADA2: high-resolution sample inference from Illumina amplicon data. Nat Methods. 2016;13:581–3. 27214047 10.1038/nmeth.3869 PMC4927377

[bib10] Caron DA , Davis PG, Madin LP et al. Heterotrophic bacteria and bacteriovorous protozoa in oceanic macroaggregates. Science. 1982;218:795–7. 17771038 10.1126/science.218.4574.795

[bib33] Crespo BG , Pommier T, Fernandez-Gomez B et al. Taxonomic composition of the particle-attached and free-living bacterial assemblages in the Northwest Mediterranean Sea analyzed by pyrosequencing of the 16S rRNA. Microbiologyopen. 2013;2:541–52. 23723056 10.1002/mbo3.92 PMC3948605

[bib68] Delmont TO , Kiefl E, Kilinc O et al. Single-amino acid variants reveal evolutionary processes that shape the biogeography of a global SAR11 subclade. Elife. 2019;8:e46497. 31478833 10.7554/eLife.46497 PMC6721796

[bib26] DeLong EF , Franks DG, Alldredge AL. Phylogenetic diversity of aggregate-attached vs free-living marine bacterial assemblages. Limnol Oceanogr. 1993;38:924–34.

[bib62] Demko AM , Patin NV, Jensen PR. Microbial diversity in tropical marine sediments assessed using culture-dependent and culture-independent techniques. Environ Microbiol. 2021;23:6859–75. 34636122 10.1111/1462-2920.15798 PMC8670064

[bib48] Eiler A , Hayakawa DH, Church MJ et al. Dynamics of the SAR11 bacterioplankton lineage in relation to environmental conditions in the oligotrophic North Pacific subtropical gyre. Environ Microbiol. 2009;11:2291–300. 19490029 10.1111/j.1462-2920.2009.01954.x

[bib8] Eilers H , Pernthaler J, Glockner FO et al. Culturability and *in situ* abundance of pelagic bacteria from the North Sea. Appl Environ Microbiol. 2000;66:3044–51. 10877804 10.1128/aem.66.7.3044-3051.2000 PMC92109

[bib40] Frank JA , Reich CI, Sharma S et al. Critical evaluation of two primers commonly used for amplification of bacterial 16S rRNA genes. Appl Environ Microbiol. 2008;74:2461–70. 18296538 10.1128/AEM.02272-07 PMC2293150

[bib57] Gerdts G , Wichels A, Döpke H et al. 40-year long-term study of microbial parameters near Helgoland (German Bight, North Sea): historical view and future perspectives. Helgol Mar Res. 2004;58:230–42.

[bib72_1671700984807] Ghiglione JF , Mevel G, Pujo-Pay M et al. Diel and seasonal variations in abundance, activity, and community structure of particle-attached and free-living bacteria in NW Mediterranean Sea. Microb Ecol. 2007;54:217–31. 17345139 10.1007/s00248-006-9189-7

[bib21] Giovannoni SJ , Thrash C, Temperton B. Implications of streamlining theory for microbial ecology. ISME J. 2014;8:1553–65. 24739623 10.1038/ismej.2014.60 PMC4817614

[bib74_1671701500012] Grossart H-P , Riemann L, Azam F. Bacterial motility in the sea and its ecological implications. Aquat Microb Ecol. 2001;25:247–58.

[bib27] Grossart HP , Tang KW, Kiorboe T et al. Comparison of cell-specific activity between free-living and attached bacteria using isolates and natural assemblages. FEMS Microbiol Lett. 2007;266:194–200. 17233730 10.1111/j.1574-6968.2006.00520.x

[bib51] Gu ZG , Eils R, Schlesner M. Complex heatmaps reveal patterns and correlations in multidimensional genomic data. Bioinformatics. 2016;32:2847–9. 27207943 10.1093/bioinformatics/btw313

[bib52] Gu ZG , Gu L, Eils R et al. circlize implements and enhances circular visualization in R. Bioinformatics. 2014;30:2811–2. 24930139 10.1093/bioinformatics/btu393

[bib31] Hahn MW , Stadler P, Wu QL et al. The filtration–acclimatization method for isolation of an important fraction of the not readily cultivable bacteria. J Microbiol Methods. 2004;57:379–90. 15134885 10.1016/j.mimet.2004.02.004

[bib5] Hahnke RL , Bennke CM, Fuchs BM et al. Dilution cultivation of marine heterotrophic bacteria abundant after a spring phytoplankton bloom in the North Sea. Environ Microbiol. 2015;17:3515–26. 24725270 10.1111/1462-2920.12479

[bib9] Hahnke RL , Harder J. Phylogenetic diversity of flavobacteria isolated from the North Sea on solid media. Syst Appl Microbiol. 2013;36:497–504. 23957959 10.1016/j.syapm.2013.06.006

[bib58] Heins A , Amann RI, Harder J. Cultivation of particle-associated heterotrophic bacteria along a spring phytoplankton bloom in the North Sea. Syst Appl Microbiol. 2021a;44. 10.1016/j.syapm.2021.126232. 34399113

[bib35] Heins A , Reintjes G, Amann RI et al. Particle collection in Imhoff sedimentation cones enriches both motile chemotactic and particle-attached bacteria. Front Microbiol. 2021b;12:1–17. 10.3389/fmicb.2021.643730 PMC8047139 33868201

[bib36] Iversen MH , Ploug H. Ballast minerals and the sinking carbon flux in the ocean: carbon-specific respiration rates and sinking velocity of marine snow aggregates. Biogeosciences. 2010;7:2613–24.

[bib2] Joint I , Mühling M, Querellou J. Culturing marine bacteria - an essential prerequisite for biodiscovery. Microb Biotechnol. 2010;3:564–75. 21255353 10.1111/j.1751-7915.2010.00188.x PMC3815769

[bib54] Juggins S . rioja: Analysis of quaternary science data. 2020. *R package version (0.9-26)* [Online]. Available: cran.r-project.org/package=rioja (July 2021, date last accessed).

[bib61] Junkins EN , Stevenson BS. Using plate-Wash PCR and high-Throughput sequencing to measure cultivated diversity for natural product discovery efforts. Front Microbiol. 2021;12:675798. 34354680 10.3389/fmicb.2021.675798 PMC8329497

[bib20] Kappelmann L , Krüger K, Hehemann J-H et al. Polysaccharide utilization loci of North Sea *flavobacteriia* as basis for using SusC/D-protein expression for predicting major phytoplankton glycans. ISME J. 2019;13:76–91. 30111868 10.1038/s41396-018-0242-6 PMC6298971

[bib53] Kembel SW , Cowan PD, Helmus MR et al. Picante: r tools for integrating phylogenies and ecology. Bioinformatics. 2010;26:1463–4. 20395285 10.1093/bioinformatics/btq166

[bib12] Koch AL . Oligotrophs versus copiotrophs. Bioessays. 2001;23:657–61. 11462219 10.1002/bies.1091

[bib14] Lauro FM , McDougald D, Thomas T et al. The genomic basis of trophic strategy in marine bacteria. Proc Natl Acad Sci. 2009;106:15527–33. 19805210 10.1073/pnas.0903507106 PMC2739866

[bib24] Liao C , Liang X, Soupir ML et al. Cellular, particle and environmental parameters influencing attachment in surface waters: a review. J Appl Microbiol. 2015;119:315–30. 26033178 10.1111/jam.12860

[bib64] Liu Y , Lai Q, Shao Z. Proposal for transfer of *oceanibulbus indolifex* Wagner-Döbler et al. 2004 to the genus *sulfitobacter* as *sulfitobacter indolifex* comb. nov. Int J Syst Evol Microbiol. 2017;67:2328–31. 28693681 10.1099/ijsem.0.001950

[bib41] Ludwig W , Viver T, Westram R et al. Release LTP_12_2020, featuring a new ARB alignment and improved 16S rRNA tree for prokaryotic type strains. Syst Appl Microbiol. 2021;44:126218. 34111737 10.1016/j.syapm.2021.126218

[bib59] Mestre M , Ferrera I, Borrull E et al. Spatial variability of marine bacterial and archaeal communities along the particulate matter continuum. Mol Ecol. 2017;26:6827–40. 29117634 10.1111/mec.14421

[bib73_1671701247717] Mitchell JG , Pearson L, Bonazinga A et al. Long lag times and high velocities in the motility of natural assemblages of marine bacteria. Appl Environ Microbiol. 1995;61:877–82. 16534971 10.1128/aem.61.3.877-882.1995 PMC1388370

[bib13] Morris RM , Rappé MS, Connon SA et al. SAR11 clade dominates ocean surface bacterioplankton communities. Nature. 2002;420:806–10. 12490947 10.1038/nature01240

[bib39] Muyzer G , Teske A, Wirsen CO et al. Phylogenetic relationships of *Thiomicrospira* species and their identification in deep-sea hydrothermal vent samples by denaturing gradient gel electrophoresis of 16S rDNA fragments. Arch Microbiol. 1995;164:165–72. 7545384 10.1007/BF02529967

[bib47] Oksanen J , Blanchet FG, Friendly M et al. Vegan: Community ecology package. 2020. *R Package version 2.5-7* [Online]. Available: CRAN.R-project.org/package=vegan (July 2021, date last accessed).

[bib7] Oppenheimer CH , ZoBell CE. The growth and viability of sixty-three species of marine bacteria as influenced by hydrostatic pressure. J Mar Res. 1952;11:10–8.

[bib66] Oyola SO , Otto TD, Gu Y et al. Optimizing illumina next-generation sequencing library preparation for extremely at-biased genomes. BMC Genomics. 2012;13:1. 22214261 10.1186/1471-2164-13-1 PMC3312816

[bib30] Pedler BE , Aluwihare LI, Azam F. Single bacterial strain capable of significant contribution to carbon cycling in the surface ocean. Proc Natl Acad Sci. 2014;111:7202–7. 24733921 10.1073/pnas.1401887111 PMC4034236

[bib42] Pruesse E , Peplies J, Glöckner FO. SINA: accurate high-throughput multiple sequence alignment of ribosomal RNA genes. Bioinformatics. 2012;28:1823–9. 22556368 10.1093/bioinformatics/bts252 PMC3389763

[bib45] Quast C , Pruesse E, Yilmaz P et al. The SILVA ribosomal RNA gene database project: improved data processing and web-based tools. Nucleic Acids Res. 2013;41:590–6. 10.1093/nar/gks1219 PMC3531112 23193283

[bib71] R Core Team . R: A language and environment for statistical computing. R Foundation for Statistical Computing, Vienna, Austria; 2014.

[bib16] Rappé MS , Connon SA, Vergin KL et al. Cultivation of the ubiquitous SAR11 marine bacterioplankton clade. Nature. 2002;418:630–3. 12167859 10.1038/nature00917

[bib19] Rieck A , Herlemann DPR, Jürgens K et al. Particle-associated differ from free-living bacteria in surface waters of the Baltic Sea. Front Microbiol. 2015;6:1297. 26648911 10.3389/fmicb.2015.01297 PMC4664634

[bib44] Schloss PD , Westcott SL, Ryabin T et al. Introducing mothur: open-source, platform-independent, community-supported software for describing and comparing microbial communities. Appl Environ Microbiol. 2009;75:7537–41. 19801464 10.1128/AEM.01541-09 PMC2786419

[bib60] Seymour JR , Amin SA, Raina J-B et al. Zooming in on the phycosphere: the ecological interface for phytoplankton-bacteria relationships. Nat Microbiol. 2017;2:17065. 28555622 10.1038/nmicrobiol.2017.65

[bib46] SILVAngs . rDNA-based microbial community analysis using next-generation sequencing (NGS) data. User Guide. 2015. [Online]. (July 2021, date last accessed).

[bib17] Simon M , Grossart HP, Schweitzer B et al. Microbial ecology of organic aggregates in aquatic ecosystems. Aquat Microb Ecol. 2002;28:175–211.

[bib25] Smith DC , Simon M, Alldredge AL et al. Intense hydrolytic enzyme-activity on marine aggregates and implications for rapid particle dissolution. Nature. 1992;359:139–42.

[bib69] Smith DP , Thrash JC, Nicora CD et al. Proteomic and transcriptomic analyses of “*Candidatus* Pelagibacter ubique” describe the first PII-independent response to nitrogen limitation in a free-living *alphaproteobacterium*. Mbio. 2013a;4:e00133. 24281717 10.1128/mBio.00133-12 PMC3870248

[bib18] Smith M , Zeigler Allen L, Allen A et al. Contrasting genomic properties of free-living and particle-attached microbial assemblages within a coastal ecosystem. Front Microbiol. 2013b;4:120. 23750156 10.3389/fmicb.2013.00120 PMC3668451

[bib1] Staley JT , Konopka A. Measurement of in situ activities of nonphotosynthetic microorganisms in aquatic and terrestrial habitats. Annu Rev Microbiol. 1985;39:321–46. 3904603 10.1146/annurev.mi.39.100185.001541

[bib3] Steen AD , Crits-Christoph A, Carini P et al. High proportions of bacteria and archaea across most biomes remain uncultured. ISME J. 2019;13:3126–30. 31388130 10.1038/s41396-019-0484-y PMC6863901

[bib22] Stocker R , Seymour JR. Ecology and physics of bacterial chemotaxis in the ocean. Microbiol Mol Biol Rev. 2012;76:792–812. 23204367 10.1128/MMBR.00029-12 PMC3510523

[bib11] Stocker R . Marine microbes see a sea of gradients. Science. 2012;338:628–33. 23118182 10.1126/science.1208929

[bib49] Teeling H , Fuchs BM, Becher D et al. Substrate-controlled succession of marine bacterioplankton populations induced by a phytoplankton bloom. Science. 2012;336:608–11. 22556258 10.1126/science.1218344

[bib50] Teeling H , Fuchs BM, Bennke CM et al. Recurring patterns in bacterioplankton dynamics during coastal spring algae blooms. Elife. 2016;5:e11888. 27054497 10.7554/eLife.11888 PMC4829426

[bib56] Wickham H , François R, Henry L et al. dplyr: A grammar of data manipulation. 2018. *R package version 0.7.8* [Online]. Available: CRAN.R-project.org/package=dplyr (July 2021, date last accessed).

[bib37] Winkelmann N , Harder J. An improved isolation method for attached-living *planctomycetes* of the genus *Rhodopirellula*. J Microbiol Methods. 2009;77:276–84. 19303037 10.1016/j.mimet.2009.03.002

[bib63] Wirth JS , Whitman WB. Phylogenomic analyses of a clade within the roseobacter group suggest taxonomic reassignments of species of the genera *Aestuariivita, Citreicella, Loktanella, Nautella, Pelagibaca, Ruegeria, Thalassobius, Thiobacimonas* and *Tropicibacter*, and the proposal of six novel genera. Int J Syst Evol Microbiol. 2018;68:2393–411. 29809121 10.1099/ijsem.0.002833

[bib55] Zeileis A , Hornik K, Murrell P. Escaping RGBland: selecting colors for statistical graphics. Comp Stat Data Anal. 2009;53:3259–70.

[bib28] Ziervogel K , Arnosti C. Polysaccharide hydrolysis in aggregates and free enzyme activity in aggregate-free seawater from the north-eastern Gulf of Mexico. Environ Microbiol. 2008;10:289–99. 18093165 10.1111/j.1462-2920.2007.01451.x

[bib29] Ziervogel K , Steen AD, Arnosti C. Changes in the spectrum and rates of extracellular enzyme activities in seawater following aggregate formation. Biogeosciences. 2010;7:1007–15.

[bib67] Žure M , Fernandez-Guerra A, Munn CB et al. Geographic distribution at subspecies resolution level: closely related *Rhodopirellula* species in European coastal sediments. ISME J. 2017;11:478–89. 27801907 10.1038/ismej.2016.123 PMC5270564

