# Supplementary material for: Particle-associated bacteria in seawater dominate the colony-forming microbiome on ZoBell marine agar
Source: FEMS Microbiol Ecol. 2022 Dec 13;99(1):fiac151. doi: 10.1093/femsec/fiac151 (PMC9798892; doi:10.1093/femsec/fiac151)
Supplement: fiac151_Supplemental_Files [file fiac151_supplemental_files.zip › Supp_data_AHE_2_220727.pdf]

Supplemental Material

Table S1. Calculated cultivability in percent, based on counts of particle-attached cell. Samples were taken during a phytoplankton Spring bloom off Helgoland in 2018. Particle cell counts were taken from (Heins et al., 2021).

| | 12.4.18 | 18.4.18 | 25.4.18 | 08.5.18 | 22.5.18 |
|---|---|---|---|---|---|
| F_0.2µm | 0.5 | 0.4 | 0.8 | 0.9 | 1.0 |
| F_3µm | 3.4 | 2.6 | 10.0 | 5.1 | 8.2 |
| F_10µm | 66.0 | 41.0 | 25.2 | 12.4 | 14.8 |
| C_FL | nd | 0.5 | 0.4 | nd | nd |
| C_PA | nd | 1.5 | 0.7 | nd | nd |
| SC_TF | nd | 0.5 | 0.8 | 0.9 | 1.2 |
| SC_BF | 0.9 | 26.3 | 12.5 | 3.9 | 10.1 |
| SC_BF_FL | nd | n | nd | nd | nd |
| SC_BF_PA | nd | n | 117.2 | nd | nd |

F_0.2µm, F_3µm, F_10µm: sequentially filtered fractions of the respective size range 3-0.2 µm, 10-3 µm and >10 µm; C_FL/PA: centrifugation-derived free-living (= supernatant) and particle-attached (= pellet) fraction; SC_TF/BF: sedimentation cone top and bottom fraction after 3 h of gravitational settlement; SC_BF_FL/PA: sedimentation cone bottom fraction after 3 h of sedimentation, resuspension and separation by centrifugation. nd: not determined.

Table S2. Total number of strains identified using Sanger sequencing and grown on plate medium inoculated with unprocessed and fractionated seawater off Helgoland (North Sea) during a phytoplankton spring bloom in 2018.

| Genus affiliation of CFU | F_0.2µm (n = 5) | SC_TF (n = 6) | C_FL (n = 2) | SW (n = 4) | F_3µm (n = 5) | F_10µm (n = 5) | PN_20µm (n = 5) | PN_80µm (n = 5) | SC_BF (n = 10) | C_PA (n = 2) |
|---|---|---|---|---|---|---|---|---|---|---|
| *Alcanivorax* | 1 | 0 | 2 | 0 | 1 | 5 | 0 | 0 | 0 | 0 |
| *Glaciecola* | 0 | 1 | 0 | 0 | 0 | 0 | 0 | 0 | 0 | 0 |
| *Paraglaciecola* | 0 | 0 | 1 | 0 | 1 | 1 | 0 | 0 | 0 | 0 |
| *Alteromonadaceae* | 0 | 1 | 0 | 0 | 0 | 0 | 0 | 0 | 4 | 0 |
| *Arcobacter* | 2 | 7 | 1 | 6 | 1 | 0 | 0 | 1 | 0 | 0 |
| *Bacillus* | 1 | 0 | 0 | 0 | 0 | 0 | 0 | 0 | 0 | 0 |
| *Acidovorax* | 0 | 0 | 0 | 0 | 1 | 0 | 0 | 0 | 0 | 0 |
| *Limnobacter* | 0 | 0 | 0 | 0 | 0 | 0 | 0 | 3 | 0 | 0 |
| *Verticia* | 0 | 0 | 0 | 0 | 0 | 1 | 0 | 0 | 0 | 0 |
| *Brevundimonas* | 0 | 0 | 0 | 1 | 0 | 0 | 0 | 0 | 1 | 0 |
| *Colwellia* | 0 | 1 | 1 | 0 | 5 | 1 | 0 | 2 | 0 | 0 |
| *Colwelliaceae* | 0 | 0 | 0 | 0 | 0 | 0 | 2 | 0 | 0 | 0 |
| *Cryomorphaceae* u.c. | 0 | 0 | 0 | 0 | 0 | 0 | 1 | 0 | 0 | 0 |
| *Algoriphagus* | 0 | 0 | 0 | 0 | 0 | 1 | 0 | 0 | 0 | 0 |
| *Cyclobacterium* | 0 | 0 | 0 | 0 | 0 | 0 | 0 | 0 | 0 | 0 |
| *Polaribacter huanghezhanensis* | 0 | 0 | 0 | 0 | 0 | 0 | 1 | 0 | 0 | 0 |
| *Algibacter* | 2 | 0 | 3 | 2 | 0 | 2 | 1 | 0 | 0 | 1 |
| *Aquibacter* | 0 | 0 | 0 | 0 | 0 | 0 | 0 | 0 | 1 | 0 |
| *Arenibacter* | 0 | 0 | 0 | 0 | 0 | 1 | 0 | 0 | 0 | 0 |
| *Cellulophaga* | 0 | 0 | 0 | 3 | 0 | 8 | 2 | 2 | 8 | 0 |
| *Dokdonia* | 0 | 0 | 0 | 0 | 0 | 0 | 1 | 0 | 7 | 0 |
| *Flavobacterium* | 1 | 6 | 3 | 8 | 0 | 3 | 3 | 5 | 6 | 1 |
| *Gaetbulibacter* | 0 | 0 | 0 | 1 | 0 | 1 | 0 | 0 | 0 | 0 |
| *Gramella* | 0 | 0 | 0 | 0 | 0 | 0 | 2 | 2 | 0 | 0 |
| *Kordia* | 0 | 0 | 0 | 0 | 1 | 0 | 0 | 0 | 0 | 0 |
| *Leeuwenhoekiella* | 1 | 0 | 0 | 0 | 0 | 0 | 0 | 0 | 0 | 0 |

| | | | | | | | | | | |
|---|---|---|---|---|---|---|---|---|---|---|
| *Lutibacter* | 0 | 0 | 0 | 1 | 1 | 1 | 1 | 0 | 0 | 0 |
| *Lutimonas* | 0 | 0 | 0 | 0 | 1 | 1 | 0 | 0 | 0 | 1 |
| *Maribacter* | 0 | 0 | 1 | 1 | 0 | 0 | 1 | 0 | 3 | 1 |
| *Maritimimonas* | 1 | 0 | 0 | 1 | 0 | 0 | 1 | 0 | 0 | 0 |
| *Marixanthomonas* | 0 | 0 | 0 | 0 | 1 | 0 | 0 | 0 | 0 | 0 |
| *Mesonia* | 0 | 0 | 0 | 0 | 0 | 0 | 1 | 0 | 0 | 0 |
| *Muricauda* | 0 | 0 | 0 | 0 | 2 | 1 | 0 | 0 | 0 | 0 |
| *Nonlabens* | 0 | 0 | 0 | 0 | 0 | 0 | 3 | 0 | 0 | 0 |
| *Olleya* | 0 | 0 | 0 | 0 | 0 | 0 | 0 | 0 | 7 | 0 |
| *Polaribacter* | 2 | 1 | 5 | 7 | 2 | 2 | 3 | 2 | 0 | 0 |
| *Polaribacter 4* | 0 | 0 | 0 | 0 | 1 | 0 | 0 | 0 | 0 | 0 |
| *Pseudofulvibacter* | 0 | 0 | 0 | 1 | 0 | 0 | 0 | 0 | 0 | 0 |
| *Salegentibacter* | 0 | 0 | 0 | 0 | 0 | 0 | 0 | 1 | 0 | 0 |
| *Sediminicola* | 0 | 0 | 1 | 1 | 0 | 0 | 0 | 0 | 0 | 0 |
| *Subsaxibacter* | 0 | 0 | 0 | 1 | 0 | 0 | 0 | 0 | 0 | 0 |
| *Tenacibaculum* | 1 | 0 | 2 | 1 | 0 | 1 | 0 | 4 | 0 | 0 |
| *Ulvibacter* | 0 | 1 | 0 | 0 | 0 | 0 | 0 | 0 | 0 | 0 |
| *Winogradskyella* | 2 | 1 | 0 | 0 | 0 | 7 | 2 | 2 | 1 | 1 |
| *Flavobacteriaceae* | 10 | 6 | 2 | 3 | 16 | 7 | 7 | 1 | 2 | 3 |
| *Halomonas* | 6 | 0 | 0 | 0 | 3 | 0 | 0 | 1 | 0 | 0 |
| *Hyphomonas* | 0 | 1 | 0 | 0 | 0 | 1 | 0 | 0 | 0 | 0 |
| *Maricaulis* | 0 | 0 | 1 | 0 | 0 | 1 | 0 | 0 | 0 | 0 |
| *Hyphomonadaceae* | 3 | 0 | 1 | 0 | 0 | 0 | 0 | 0 | 0 | 0 |
| *Magnetospira* | 0 | 0 | 1 | 0 | 0 | 0 | 0 | 0 | 0 | 0 |
| *Marinobacter* | 0 | 0 | 0 | 0 | 0 | 0 | 1 | 0 | 0 | 0 |
| *Marinomonas* | 0 | 2 | 1 | 0 | 0 | 0 | 0 | 0 | 0 | 0 |
| *Kocuria* | 1 | 0 | 0 | 0 | 0 | 0 | 0 | 0 | 0 | 0 |
| *Micrococcus* | 0 | 0 | 0 | 0 | 0 | 0 | 0 | 0 | 0 | 1 |
| *Psychrobacter* | 0 | 1 | 0 | 0 | 0 | 6 | 5 | 3 | 5 | 0 |
| *Amphritea* | 0 | 2 | 0 | 0 | 0 | 0 | 0 | 0 | 0 | 0 |
| *Neptunomonas* | 2 | 0 | 0 | 1 | 0 | 0 | 1 | 0 | 0 | 0 |

| | | | | | | | | | | |
|---|---|---|---|---|---|---|---|---|---|---|
| *Profundimonas* | 0 | 0 | 0 | 0 | 0 | 1 | 0 | 0 | 0 | 0 |
| *Rhodococcus* | 1 | 2 | 1 | 4 | 4 | 0 | 1 | 0 | 0 | 0 |
| *Nocardioides* | 1 | 0 | 0 | 0 | 0 | 0 | 1 | 1 | 0 | 0 |
| NS7 marine group | 0 | 0 | 0 | 0 | 0 | 0 | 0 | 0 | 0 | 1 |
| *Sunxiuqinia* | 1 | 0 | 0 | 0 | 0 | 0 | 0 | 0 | 0 | 0 |
| *Pseudoalteromonas* | 2 | 10 | 1 | 5 | 7 | 12 | 9 | 5 | 10 | 4 |
| *Pseudoalteromonadaceae* | 0 | 1 | 0 | 0 | 1 | 0 | 0 | 0 | 0 | 0 |
| *Pseudomonas* | 0 | 0 | 0 | 1 | 0 | 0 | 4 | 1 | 0 | 0 |
| *Psychromonas* | 5 | 1 | 0 | 5 | 6 | 2 | 1 | 4 | 0 | 0 |
| *Cerasicoccus* | 0 | 1 | 0 | 0 | 0 | 0 | 0 | 0 | 0 | 0 |
| *Ahrensia* | 0 | 0 | 0 | 0 | 1 | 0 | 0 | 0 | 0 | 0 |
| *Aurantimonas* | 0 | 0 | 0 | 0 | 0 | 0 | 2 | 4 | 0 | 0 |
| *Hoeflea* | 5 | 0 | 0 | 2 | 2 | 0 | 0 | 0 | 0 | 0 |
| *Anderseniella* | 0 | 1 | 0 | 0 | 0 | 0 | 0 | 0 | 0 | 0 |
| *Amylibacter* | 0 | 0 | 0 | 0 | 0 | 1 | 0 | 0 | 0 | 0 |
| *Celeribacter* | 0 | 1 | 0 | 0 | 0 | 0 | 0 | 0 | 0 | 0 |
| *Ketogulonicigenium* | 0 | 0 | 0 | 0 | 0 | 0 | 1 | 3 | 0 | 0 |
| *Lentibacter* | 4 | 2 | 2 | 4 | 0 | 1 | 1 | 0 | 1 | 2 |
| *Litoreibacter* | 0 | 0 | 0 | 0 | 0 | 0 | 1 | 0 | 0 | 1 |
| *Loktanella* | 1 | 2 | 0 | 1 | 0 | 2 | 1 | 0 | 5 | 0 |
| *Paracoccus* | 0 | 0 | 0 | 0 | 0 | 1 | 1 | 0 | 0 | 0 |
| *Planktomarina* | 8 | 10 | 1 | 6 | 3 | 1 | 1 | 0 | 3 | 1 |
| *Planktotalea* | 1 | 3 | 0 | 0 | 0 | 0 | 0 | 0 | 1 | 0 |
| *Pseudophaeobacter* | 0 | 0 | 2 | 2 | 2 | 0 | 0 | 0 | 0 | 0 |
| Roseobacter clade NAC11-7 lineage | 1 | 0 | 0 | 0 | 0 | 0 | 0 | 0 | 0 | 0 |
| *Roseovarius* | 0 | 0 | 1 | 2 | 1 | 0 | 0 | 0 | 0 | 0 |
| *Ruegeria* | 0 | 0 | 0 | 0 | 0 | 0 | 1 | 0 | 0 | 0 |
| *Sulfitobacter* | 9 | 12 | 0 | 10 | 11 | 18 | 17 | 12 | 20 | 2 |
| *Rhodobacteraceae* u.c. | 2 | 1 | 0 | 0 | 2 | 1 | 1 | 0 | 0 | 0 |
| *Rhodobacteraceae* | 2 | 2 | 1 | 1 | 1 | 2 | 4 | 1 | 2 | 0 |

| | | | | | | | | | | |
|---|---|---|---|---|---|---|---|---|---|---|
| *Thalassolituus* | 0 | 1 | 0 | 0 | 0 | 0 | 0 | 0 | 0 | 0 |
| *Salinisphaera* | 0 | 0 | 0 | 0 | 1 | 0 | 2 | 1 | 0 | 0 |
| *Lewinella* | 0 | 0 | 0 | 0 | 0 | 0 | 0 | 1 | 0 | 0 |
| *Psychrobium* | 0 | 0 | 0 | 1 | 0 | 0 | 0 | 0 | 0 | 0 |
| *Shewanella* | 2 | 0 | 2 | 0 | 0 | 0 | 1 | 0 | 3 | 2 |
| *Sneathiella* | 2 | 1 | 0 | 0 | 0 | 0 | 0 | 0 | 0 | 0 |
| *Altererythrobacter* | 0 | 0 | 0 | 1 | 0 | 0 | 0 | 0 | 0 | 0 |
| *Erythrobacter* | 0 | 1 | 0 | 0 | 0 | 0 | 0 | 0 | 1 | 0 |
| *Sphingopyxis* | 0 | 3 | 0 | 0 | 0 | 0 | 0 | 0 | 0 | 0 |
| *Sphingorhabdus* | 2 | 0 | 1 | 0 | 0 | 0 | 0 | 0 | 0 | 0 |
| *Sphingomonadaceae* | 1 | 0 | 0 | 2 | 0 | 0 | 12 | 1 | 0 | 0 |
| *Spongiibacter* | 0 | 0 | 0 | 0 | 0 | 0 | 1 | 0 | 0 | 0 |
| *Zhongshania* | 0 | 0 | 1 | 0 | 0 | 0 | 1 | 0 | 0 | 1 |
| *Stappia* | 0 | 0 | 0 | 1 | 0 | 0 | 0 | 0 | 0 | 0 |
| *Terasakiella* | 0 | 0 | 0 | 0 | 0 | 1 | 0 | 0 | 0 | 0 |
| *Terasakiellaceae* u.c. | 0 | 0 | 0 | 0 | 1 | 0 | 0 | 0 | 0 | 0 |
| *Granulosicoccus* | 0 | 0 | 0 | 1 | 0 | 0 | 1 | 0 | 0 | 0 |
| *Aliivibrio* | 0 | 2 | 0 | 0 | 1 | 0 | 0 | 2 | 0 | 0 |
| *Vibrio* | 0 | 0 | 0 | 1 | 2 | 1 | 0 | 0 | 3 | 0 |

F_0.2µm, F_3µm, F_10µm: sequentially filtered fractions of the respective size range 3-0.2 µm, 10-3 µm and >10 µm; C_FL/PA: centrifugation-derived free-living (= supernatant) and particle-attached (= pellet) fraction; PN_20µm/ 80µm: plankton net fractions caught with a pore size of 20 µm or 80 µm; SC_TF/BF: sedimentation cone top and bottom fraction after 3 h of gravitational settlement; SC_BF_FL/PA: sedimentation cone bottom fraction after 3 h of sedimentation, resuspension and separation by centrifugation into a free-living (FL) and particle-attached (PA) fraction; SW: seawater. u.c. uncultured.

Table S3. Analysis of amplified sequence variants (ASVs). Relative read abundances of all ASVs in all fractions of all 2018 samples (Excel sheet, electronic supplementary material 1). Abbreviations of the file names: 20µm: plankton net fraction 20 µm, 80µm: plankton net fraction 80 µm, BF: bottom fraction, C: centrifugation, F_0.2µm: filtered fraction 3-0.2 µm, F_3µm: filtered fraction 10-3 µm, F_10µm: filtered fraction > 10 µm, FL: free-living fraction, PA: particle-attached fraction, SC: sedimentation cone, TF: top fraction. Numbers behind the sample names indicate the time of each sampling group. 1: 04.04.2018- 12.04.2018; 2: 13.04.2018-20.04.2018; 3: 23.04.2018-27.04.2018; 4: 07.05.2018- 09.05.2018; 5: 22.05.2018- 23.05.2018. EF describes the enrichment factor of the samples, obtained by dividing the relative read abundance of the sample through the relative read abundance in seawater.

Table S4. Number of harvested plates that were combined to form sample groups based on the separation method, fraction and sampling time point. Seawater was sampled and processed during a phytoplankton Spring bloom off Helgoland in 2018.

| | **SC** | | **SC_BF** | | **C** | | **F** | | | **PN** | | **SW** |
|---|---|---|---|---|---|---|---|---|---|---|---|---|
| | **TF** | **BF** | **FL** | **PA** | **FL** | **PA** | **0.2µm** | **3µm** | **10µm** | **20µm** | **80µm** | |
| **Time point 1** (04.04.2018-12.04.2018) | nd | 6 | nd | nd | nd | nd | 12 | 12 | 15 | 3 | 3 | nd |
| **Time point 2** (13.04.2018-20.04.2018) | 18 | 18 | 9 | 8 | 8 | 6 | 8 | 6 | 3 | 3 | 3 | 19 |
| **Time point 3** (23.04.2018-27.04.2018) | 14 | 17 | 10 | 8 | 8 | 15 | 16 | 18 | 15 | 6 | 6 | 15 |
| **Time point 4** (07.05.2018-09.05.2018) | 9 | 9 | nd | nd | nd | nd | 9 | 9 | 9 | 3 | 3 | 9 |
| **Time point 5** (22.05.2018-23.05.2018) | 5 | 7 | nd | nd | nd | nd | 4 | 3 | 3 | 3 | 3 | 11 |

F_0.2µm, F_3µm, F_10µm: sequentially filtered fractions of the respective size range 3-0.2 µm, 10-3 µm and >10 µm; C_FL/PA: centrifugation-derived free-living (= supernatant) and particle-attached (= pellet) fraction; PN_20µm/ 80µm: plankton net fractions caught with a pore size of 20 µm or 80 µm; SC_TF/BF: sedimentation cone top and bottom fraction after 3 h of gravitational settlement; SC_BF_FL/PA: sedimentation cone bottom fraction after 3 h of sedimentation, resuspension and separation by centrifugation into a free-living (FL) and particle-attached (PA) fraction; SW: seawater.

Table S5. PERMANOVA of unfractionated seawater and fractionated seawater using sequential filtration, centrifugation and natural gravitation in cones. Samples were obtained during a phytoplankton spring bloom off Helgoland (54°11’03”N, 7°54’00”E) in 2018.

| | Df | Sums of squares | Mean squares | F Model | $R^2$ | Pr(>F) | Significance |
|---|---|---|---|---|---|---|---|
| Dataset | 11 | 4.5598 | 0.41453 | 1.6814 | 0.28677 | 0.001 | 0.0001 |
| **Residuals** | 46 | 11.3408 | 0.24654 | 0.71323 | | | |
| **Total** | 57 | 15.9006 | 1 | | | | |

DF: degrees of freedom, n= number of samples.

Table S6. Pairwise comparisons using permutation MANOVAs on a distance matrix. Samples were obtained during a phytoplankton spring bloom off Helgoland (54°11'03"N, 7°54'00"E) in 2018.

| | C_ FL | C_ PA | F_ 10µm | F_ 3µm | F_ 0.2µm | PN_ 20µm | PN_ 80µm | SC_ BF | SC_ BF_FL | SC_ BF_PA | SC_ TF |
|---|---|---|---|---|---|---|---|---|---|---|---|
| **C_PA** | 0.894 | - | - | - | - | - | - | - | - | - | - |
| **F_10µm** | 0.597 | 0.512 | - | - | - | - | - | - | - | - | - |
| **F_3µm** | 0.369 | 0.551 | 0.202 | - | - | - | - | - | - | - | - |
| **F_0.2µm** | 0.576 | 0.748 | 0.388 | 0.666 | - | - | - | - | - | - | - |
| **PN_20µm** | 0.343 | 0.041 | 0.211 | 0.022 | 0.013 | - | - | - | - | - | - |
| **PN_80µm** | 0.21 | 0.059 | 0.211 | 0.013 | 0.013 | 0.607 | - | - | - | - | - |
| **SC_BF** | 0.176 | 0.041 | 0.202 | 0.013 | 0.013 | 0.314 | 0.492 | - | - | - | - |
| **SC_BF_FL** | 0.748 | 0.796 | 0.612 | 0.566 | 0.556 | 0.551 | 0.343 | 0.512 | - | - | - |
| **SC_BF_PA** | 0.512 | 0.388 | 0.478 | 0.264 | 0.241 | 0.551 | 0.326 | 0.551 | 1 | - | - |
| **SC_TF** | 0.6 | 0.411 | 0.838 | 0.511 | 0.796 | 0.343 | 0.202 | 0.202 | 0.551 | 0.343 | - |
| **SW** | 0.838 | 0.388 | 0.471 | 0.213 | 0.607 | 0.211 | 0.084 | 0.044 | 0.326 | 0.211 | 0.977 |

F_0.2µm, F_3µm, F_10µm: sequentially filtered fractions of the respective size range 3-0.2 µm, 10-3 µm and >10 µm; C_FL/PA: centrifugation-derived free-living (= supernatant) and particle-attached (= pellet) fraction; PN_20µm/ 80µm: plankton net fractions caught with a pore size of 20 µm or 80 µm; SC_TF/BF: sedimentation cone top and bottom fraction after 3 h of gravitational settlement; SC_BF_FL/PA: sedimentation cone bottom fraction after 3 h of sedimentation, resuspension and separation by centrifugation into a free-living (FL) and particle-attached (PA) fraction; SW: seawater.

## Reference

Heins, A., Reintjes, G., Amann, R.I., and Harder, J. (2021). Particle collection in Imhoff sedimentation cones enriches both motile chemotactic and particle-attached bacteria. *Frontiers in Microbiology* 12(619), 1-17. doi: 10.3389/fmicb.2021.643730.
